# Current Management of In-Stent Restenosis

**DOI:** 10.3390/jcm13082377

**Published:** 2024-04-19

**Authors:** Daniele Giacoppo, Placido Maria Mazzone, Davide Capodanno

**Affiliations:** Division of Cardiology, Azienda Ospedaliero-Universitaria Policlinico “Rodolico—San Marco”, Department of Surgery and Medical-Surgical Specialties, University of Catania, via Santa Sofia 78, 95124 Catania, Italydcapodanno@unict.it (D.C.)

**Keywords:** in-stent restenosis, percutaneous coronary intervention, drug-coated balloon, drug-eluting stent, intravascular imaging

## Abstract

In-stent restenosis (ISR) remains the primary cause of target lesion failure following percutaneous coronary intervention (PCI), resulting in 10-year incidences of target lesion revascularization at a rate of approximately 20%. The treatment of ISR is challenging due to its inherent propensity for recurrence and varying susceptibility to available strategies, influenced by a complex interplay between clinical and lesion-specific conditions. Given the multiple mechanisms contributing to the development of ISR, proper identification of the underlying substrate, especially by using intravascular imaging, becomes pivotal as it can indicate distinct therapeutic requirements. Among standalone treatments, drug-coated balloon (DCB) angioplasty and drug-eluting stent (DES) implantation have been the most effective. The main advantage of a DCB-based approach is the avoidance of an additional metallic layer, which may otherwise enhance neointimal hyperplasia, provide the substratum for developing neoatherosclerosis, and expose the patient to a persistently higher risk of coronary ischemic events. On the other hand, target vessel scaffolding by DES implantation confers relevant mechanical advantages over DCB angioplasty, generally resulting in larger luminal gain, while drug elution from the stent surface ensures the inhibition of neointimal hyperplasia. Nevertheless, repeat stenting with DES also implies an additional permanent metallic layer that may reiterate and promote the mechanisms leading to ISR. Against this background, the selection of either DCB or DES on a patient- and lesion-specific basis as well as the implementation of adjuvant treatments, including cutting/scoring balloons, intravascular lithotripsy, and rotational atherectomy, hold the potential to improve the effectiveness of ISR treatment over time. In this review, we comprehensively assessed the available evidence from randomized trials to define contemporary interventional treatment of ISR and provide insights for future directions.

## 1. Introduction

In-stent restenosis (ISR) is a primary determinant of long-term percutaneous coronary intervention (PCI) failure and is traditionally defined as an angiographic reduction of ≥50% of the luminal diameter within a previously implanted stent or 5 mm segments proximally or distally (“stent edges”) of a previously implanted stent [1,2,3].

Before the advent of stents, plain balloon angioplasty was associated with 6-month restenosis (i.e., not ISR) at an incidence of up to 60%, predominantly due to acute elastic recoil and vascular remodelling [4,5,6]. The introduction of bare metal stents (BMSs) led to more predictable outcomes and reduced restenosis (i.e., ISR). Nevertheless, 1-year incidences of ISR remained around 20–30%, mainly due to exaggerated neointimal proliferation 3–6 months following BMS implantation-related vascular wall damage and stent endothelialization, which consequently leads to the development of a pro-inflammatory environment that promotes the recruitment and activation of fibroblasts [6,7]. Drug-eluting stents (DESs), through the integration of a metallic stent platform with the release of an antiproliferative medication, have substantially reduced ISR at 1-year incidences ranging from 2 to 4% to approximately 10%, depending on the individual risk profile and coronary artery disease complexity [8,9]. However, although the release of antiproliferative drugs efficiently mitigates the mechanisms of ISR, several studies have shown delayed mechanisms of neointimal proliferation, neoatherosclerosis, and local hypersensitivity reactions leading to persistently significant incidences of ISR [10,11,12]. In particular, the 10-year data from randomized trials with DES show incidences of target lesion revascularization mostly due to an ISR of about 20% [13,14].

Against this background, considering the improved life expectancy of patients and the contemporary treatment of more challenging coronary artery disease patterns, ISR remains the most important and frequent adverse event following PCI despite technical improvements in contemporary DES design, drugs, and polymers [15,16]. The purpose of this review is to provide a comprehensive overview of contemporary devices and techniques for the treatment of ISR.

## 2. Classifications

**Figure 1:**
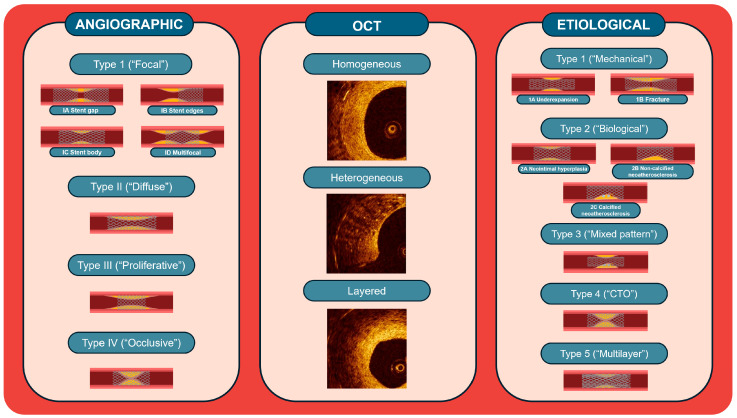
Main classifications of coronary ISR. CTO = Chronic total occlusion; OCT = optical coherence tomography.

The most widely used classification of coronary ISR is based on the angiographic assessment [17]. Four main patterns of ISR can be identified depending on the extension, location, and complete occlusion of the vessel (Figure 1): Type 1 (“Focal”) has an extension of <10 mm within and/or contiguous to the previously implanted stent, and depending on the localization, it can be further distinguished into 1A when localized in the gap between two stents, 1B when localized in the stent edges, 1C when localized in the body of a stent, and 1D when there are more Type 1 lesions (“Multifocal”); Type 2 (“Diffuse”) has an extension of >10 mm within the previously implanted stent; and Type 3 (“Proliferative”) has an extension of >10 mm beyond the margins of the previously implanted stent; Type 4 (“Occlusive”) is characterized by the complete vessel occlusion with thrombolysis in myocardial infarction flow 0 [17]. The classification was associated with an increasing trend of target lesion revascularization across ISR types [17]. However, this classification was developed in the context of the initial experience with systematic stent implantation. Consequently, the characteristics of lesions originally requiring stenting, arbitrarily imposed cut-offs between ISR types, type of restenosed stent (BMS-ISR), and interventional devices employed for the treatment of BMS-ISR do not seem to apply to contemporary PCI and may have substantially influenced the prognostic implications of this classification. In addition, coronary angiography provides only a two-dimensional visualization of the vessel, potentially leading to the inappropriate estimation of ISR severity and characteristics, and it often fails to elucidate the underlying mechanisms of ISR.

Optical coherence tomography (OCT) was used to classify ISR with the identification of four groups based on the structure of the restenotic tissue (Figure 1): “Homogeneous”, characterized by uniform high signal intensity and low back-scatter, typical of areas such as smooth muscle cells; “Heterogeneous”, characterized by mixed signal intensity, potentially indicative of proteoglycan-rich neointima and early neoatherosclerosis; and “Layered”, characterized by superficial high signal intensity and deep low signal intensity, frequently in peri-strut areas. Other qualitative parameters for evaluation are the restenotic tissue back-scatter (high or low), in which the shape of the lumen can be regular or irregular, with tissue protrusion into the lumen, the presence of intraluminal material, and visible microvessels [18].

More recently, Waksman et al. proposed a classification system for DES-ISR based on the underlying mechanism responsible for ISR to guide therapeutic decisions (Figure 1) [19]. This classification encompasses the following categories: Type I (“Mechanical’) includes stent underexpansion (IA), which necessitates a high-pressure balloon with or without additional treatments such as intravascular lithotripsy, and stent fracture (IB), which necessitates further stent implantation; Type II (“Biological”), including neointimal hyperplasia (IIA) and non-calcific neoatherosclerosis (IIB), for which the optimal treatment may involve the use of a drug-coated balloon (DCB) or a DES, and calcific neoatherosclerosis (IIC), for which more aggressive interventions involving the use of a cutting/scoring balloon, rotational atherectomy, intravascular lithotripsy, or excimer laser coronary atherectomy may be required; Type III (“Mixed”) integrates mechanical and biological causes, thereby necessitating a combined treatment approach involving dilation with a high-pressure balloon followed by the implantation of DES or DCB; Type IV (“Chronic total occlusions”) requires different invasive strategies or coronary artery bypass grafting; finally, Type V (“Multiple DES-ISR”) includes >2 layers of stents implanted and implies strategies avoiding further stent layering, hence favouring the use of DCB with or without other additional treatments or coronary artery bypass grafting [19].

## 3. Mechanisms of in-Stent Restenosis

ISR is a gradual process that sometimes begins within days after stent implantation and at other times several months or years later [20,21,22,23]. Several non-mutually exclusive biological, mechanical, and patient-related mechanisms can configure ISR [20,21,22,23]. Nevertheless, differences in the composition of lesions and their timing of development often reflect the predominance of one process over the others [20,21,22,23]. Consistently, the spectrum of clinical presentations of ISR is broad, ranging from the absence of symptoms to acute coronary syndrome (ACS) [1,2,3].

While elastic recoil and vascular remodelling are the main mechanisms of restenosis following plain balloon angioplasty (i.e., non-ISR), their role in the development of ISR is very limited [5,24,25]. After stenting, endothelial denudation, vessel tissue layers stretching, and sometimes medio-intimal dissection promote fibrinogen and platelets deposition, smooth muscle cell proliferation and migration, cytokines release and recruitment of leukocytes and macrophages, and finally, extracellular matrix transformation with protein degradation and resynthesis [5,24,25,26,27]. Over the first months, these mechanisms lead to neointimal proliferation and thickening [20,21,22,23]. Although antiproliferative properties of DES significantly reduce the occurrence of ISR by contrasting an exaggerated neointimal hyperplasia and an abnormal arterial wall healing, when these medications inefficiently counteract reactive neointimal hyperplasia, DES-ISR generally presents between 6 and 9 months after implantation. The process has also been linked to inflammation and hypersensitivity reactions to the alloy and polymers of DES [1,25,28]. However, the development of DES with lower strut thickness and bioresorbable or absent polymer has provided mixed results [28].

Considering the strong interindividual susceptibility to the causal mechanisms identified, DES-ISR has been linked to comorbid conditions, genetic factors, and resistance to antiproliferative medications. In more detail, recurrent ISR is more frequent in patients with diabetes and non-first ISR [29]. However, these conditions partially explain the occurrence of recurrent ISR. Treatments targeting genes implicated in the development of ISR have been tested in several preclinical studies. Nevertheless, relevant human investigations have not been conducted in the last two decades, and studies assessing associations between ISR and genetic patterns have not identified relevant targets. Data on the genes implicated in recurrent ISR are not available. Finally, the ISAR-DESIRE 2 trial randomized patients with DES-ISR to recurrent DES implantation either with the same (i.e., sirolimus-DES) or a different DES type (i.e., paclitaxel-DES), showing no significant differences between groups at 6–8-month angiography and 12-month clinical follow-up [30].

ISR with delayed development is predominantly linked to neoatherosclerosis, which is the atherosclerosis of the neointima within the stent [20,22]. Neoatherosclerosis within DES is accelerated compared with BMS and non-stented segments and can present with different histopathologic patterns, ranging from peri-strut foamy macrophage clusters to fibroatheromas with or without calcifications and necrotic cores, thin-cap fibroatheromas, and ruptured plaques associated with thrombosis, noncontiguous with the underlying native atherosclerotic plaque behind the restenosed stent [20,22,31].

## 4. Intravascular Imaging and Functional Testing

The use of intravascular imaging and invasive functional testing often offers a more insightful anatomic and mechanistic characterization of ISR lesions and guides PCI by improving the selection of the most appropriate interventional approaches [3,32,33].

The use of intravascular ultrasound (IVUS) and OCT for the treatment of stent failure is recommended by current European Society of Cardiology guidelines (Class IIa) and a focused European Association of Percutaneous Coronary Intervention scientific document [34,35]. The two techniques have different capabilities and limitations. OCT has a high spatial resolution (10–15 µm) and provides excellent visualization of the coronary artery endoluminal surface but requires the administration of contrast medium during tomographic acquisition and shows limited tissue penetration [35,36]. OCT is superior to IVUS for identifying neoatherosclerotic plaques and the presence of thrombi [35,36]. Furthermore, OCT guarantees a more detailed visualization of stent architecture and strut apposition and a reliable characterization of neointima morphology [35,36]. IVUS has a spatial resolution 10 times lower than OCT (150 µm), but it provides high tissue penetration, generally allowing for the assessment of the vessel wall, even in the presence of multiple stent layers [3,37,38]. Recently, the results of a subanalysis on complex coronary artery disease of the OCTIVUS trial comparing IVUS with OCT for guiding PCI showed that in the setting of ISR, OCT may be associated with improved 2-year clinical outcomes compared with IVUS [39,40]. However, the incremental value of intravascular imaging for the treatment of ISR warrants randomized clinical trials, and current indications essentially rely on the experts’ consensus, observational studies, and small subanalyses of randomized trials [3,35,36].

The use of coronary physiology testing, primarily fractional flow reserve (FFR) and instantaneous wave-free ratio (iFR), improves the definition of significant ISR lesions and may predict the risk of recurrent ISR based on post-PCI functional assessment [41,42]. However, there are no randomized trials or high-quality observational investigations on the use of functional testing for coronary ISR.

## 5. Percutaneous Coronary Intervention for in-Stent Restenosis

The treatment of ISR is challenging due to the different susceptibility to interventional strategies against the extremely heterogeneous spectrum of causal mechanisms and the intrinsic propensity of this type of lesion to recur over time [1,2,3]. In the last decades, several devices such as plain balloon angioplasty, rotational atherectomy, BMS implantation, and intravascular brachytherapy have been employed for the treatment of ISR, generally with unsatisfactory results [43,44]. Nevertheless, in recent years, DES implantation and DCB angioplasty have emerged as the most effective strategies in terms of angiographic and clinical outcomes, leading the current European Society of Cardiology guidelines to endorse their preferential use for the treatment of ISR (Class I) [34,43,44]. However, variable results were observed between DES implantation and DCB angioplasty in randomized clinical trials and large-scale registries as a possible result of the influence of clinical, angiographic, and technical factors as well as the causal mechanisms of ISR [45,46,47,48,49,50,51,52,53,54,55,56]. More recently, some combined strategies employing more devices have shown promising results, which warrants more evidence and more data from high-quality randomized clinical trials.

### 5.1. Drug-Eluting Stent

Repeat stenting with DES for the treatment of ISR is currently one of the most widely used therapeutic strategies for the treatment of ISR, which has proven to be highly effective [43,44]. Target segment scaffolding by DES implantation confers relevant mechanical advantages compared with non-stent-based interventional strategies. This may make this strategy particularly appropriate for cases in which ISR is characterized by focal patterns with distributions predominantly outside the stent (i.e., margins), late ISR due to diffuse neoatherosclerosis, ISR due to fracture of the previous stent, and ISR not located in vessels with small reference vessel diameters. In contrast, DES implantation should be avoided when the primary mechanism of ISR is underexpansion and when it is recurrent ISR with multiple metallic layers.

**Figure 2:**
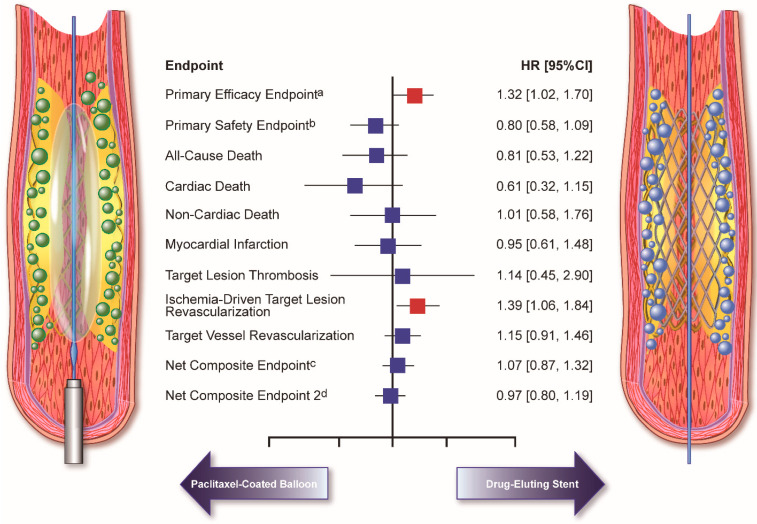
Treatment effects of DCB vs. DES for ISR from all 10 available randomized trials. CI = Confidence interval; HR = hazard ratio. ^a^ Primary efficacy endpoint: Target lesion revascularization; ^b^ Primary safety endpoint: Composite of death, myocardial infarction, or target lesion thrombosis; ^c^ Net composite endpoint 1: Composite of death, myocardial infarction, target lesion thrombosis, or target lesion revascularization; ^d^ Net composite endpoint 2: Composite of death, myocardial infarction, target lesion thrombosis, or target vessel revascularization.

In the DAEDALUS study, an individual patient meta-analysis including all 10 available randomized clinical trials comparing DCB angioplasty with DES implantation for the treatment of ISR, patients assigned to DES showed a 3-year target lesion revascularization incidence of 12.0% (Figure 2) [54]. Although there is a paucity of high-quality comparative data between new-generation DES methods for the treatment of ISR, the results of the randomized RESTENT-ISR trial showed no significant differences in the rate of major adverse cardiovascular events between everolimus- and zotarolimus-based DESs at 3-year follow-up (15.8% vs. 22.6%, *p* = 0.276) [57]. In line with these findings, the only randomized trial on ISR employing a thin-strut, bioresorbable DES (i.e., BIOLUX-RCT) showed results in line with trials based on DESs with thicker struts and durable polymers (Table 1) [30,58,59,60].

However, repeat stenting with DES also implies an additional permanent metallic layer that may reiterate and promote the mechanisms leading to ISR, primarily neointimal hyperplasia and neoatherosclerosis [72]. Indeed, although the antiproliferative medications eluted after DES implantation for de novo coronary artery disease have been associated with reduced neointimal hyperplasia and ISR compared with BMS, the rates of early recurrent ISR after repeat stenting with DES for ISR are higher compared with those observed after the treatment of de novo lesions [73,74]. In addition, late ischemic adverse events following DES implantation for ISR have sometimes shown an excess of late events compared with less effective treatments [53]. These unaddressed questions surrounding the long-term safety of repeat DES implantation for ISR warrant more data with more contemporary devices [53].

### 5.2. Drug-Coated Balloon

DCBs are generally rapid-exchange semi-compliant balloon catheters with the surface coated with an antiproliferative medication (i.e., primarily paclitaxel, more recently sirolimus or biolimus) transferred into the endothelial cells during balloon inflation [55,56,75]. Excipients (shellac, butyryl-tri-hexyl citrate, acetyl-tri-butyl citrate, resveratrol, polyethylene glycol, butylated-hydroxyl-toluene, etc.) or carriers (polymeric phospholipid nanocarriers or microspheres) binding the antiproliferative medications prevent their swift removal by blood flow, ensuring sustained presence at the treatment site, and play a crucial role in regulating their release, solubility, and absorption kinetics [55,56,76,77].

The main advantage of a DCB-based approach is the avoidance of an additional metallic layer that may further enhance exuberant neointimal hyperplasia, provide the substratum for developing neoatherosclerosis, and likely expose the patient to a persistently higher risk of coronary ischemic events [55,56,75]. DCBs have undergone testing in some randomized trials involving patients with ISR, invariably demonstrating superior effectiveness and comparable safety when compared with plain balloons (Table 1) [49,62,64,65,66,78]. The early pivotal PACCOCATH trial including patients with BMS-ISR showed that DCB angioplasty significantly reduced 6-month in-segment late lumen loss compared with plain balloon angioplasty (absolute difference 0.70, 95% CI 0.28 to 1.12; *p* = 0.003) [63]. Furthermore, DCB angioplasty decreased the incidence of major clinical events at 12 months compared with plain balloon angioplasty (4% vs. 31%, *p* = 0.01), mainly as a result of reduced target lesion revascularization (0% vs. 23%; *p* = 0.02) [63]. After the extension of the original study population to 108 patients and maximum available follow-up to 5.4 ± 1.2 years, DCB angioplasty consistently decreased major adverse cardiac events compared with plain balloon angioplasty (27.8% vs. 59.2%, *p* = 0.009), mainly driven by the reduction in target lesion revascularization (9.3% vs. 38.9%, *p* = 0.004) [74]. Later, in the ISAR-DESIRE 3 trial, 402 patients with DES-ISR were randomized at a 1:1:1 ratio to receive either balloon angioplasty. DCB angioplasty or repeat DES implantation [49]. In this trial, DCB exhibited superior anti-restenotic efficacy compared with plain balloon in terms of 6–8-month percentage diameter stenosis (38.0% vs. 54.1%, *p* < 0.0001) and reduced major adverse clinical events at 1 year (23.5% vs. 46.2%; *p* < 0.0001), predominantly driven by lower target lesion revascularization (22.1% vs. 43.5%, *p* < 0.0001) [49]. Clinical results at 3 and 10 years confirmed the significant benefit in terms of target lesion revascularization [53,79]. Consistently, Habara and colleagues conducted two randomized trials demonstrating a significant advantage of DCB over conventional balloon angioplasty in the primary endpoint of late lumen loss (0.18 ± 0.45 mm vs. 0.72 ± 0.55 mm, *p* = 0.001) and target vessel failure (6.6% vs. 31.0%; *p*< 0.001) at 6-month follow-up, respectively [62,65]. Finally, the recent results from AGENT IDE, the United States regulatory randomized trial with DCB for the treatment of DES-ISR, showed the superior clinical effectiveness of DCB compared with balloon angioplasty in reducing the rate of the composite endpoint of target lesion failure, defined as the composite of cardiac death, target vessel myocardial infarction, or ischemia-driven target lesion revascularization (17.9% vs. 28.6%; HR 0.59, 95% CI 0.42–0.84, *p* = 0.003) [66]. In terms of individual endpoints, both target lesion revascularization (13% vs. 24.7%; HR 0.50, 95% CI 0.34–0.74, *p* = 0.001) and target vessel myocardial infarction (5.8% vs. 11.1%; HR 0.51, 95% CI 0.28–0.92, *p* = 0.02) at 1 year were significantly lower in patients assigned to DCB compared with those assigned to plain balloon [66]. The results of AGENT IDE have recently led to the approval of the first DCB in the United States.

Randomized trials comparing different DCBs for ISR have generally shown comparable anti-restenotic properties [68,80,81]. However, apparently there is no class effect, as some DCBs did not meet non-inferior anti-restenotic effectiveness compared with the control [82,83]. More recently, in some small randomized clinical trials, newer sirolimus-DCBs have been compared with paclitaxel-DCBs for the treatment ISR, showing overall non-inferior angiographic and clinical results [69,83,84]. In a pooled analysis of two trials, Scheller and colleagues showed similar results between sirolimus-DCBs and paclitaxel-DCBs regarding in-segment late lumen loss at 6 months (absolute difference 0.01, 95% CI −0.23–0.24) and clinical outcomes at 12 months [69]. More recently, Han and colleagues demonstrated in a randomized trial of patients with DES-ISR (NCT04240444) the non-inferiority of sirolimus-DCBs compared with paclitaxel-DCBs in terms of 9-month in-segment late lumen loss (0.35 ± 0.47 vs. 0.31 ± 0.36; *p* = 0.82) [70].

Finally, it is worth noting that DCBs have also become an established treatment of femoropopliteal ISR due to the favourable results observed compared with plain balloons [85,86].

### 5.3. Drug-Coated Balloon vs. Drug-Eluting Stent

Two pivotal network meta-analyses compared available strategies for the treatment of ISR, such as balloon angioplasty, intravascular brachytherapy, bare metal stent implantation, rotational atherectomy, cutting balloon, DCB angioplasty, and DES implantation, and showed that the use of DCBs and DESs was associated with the highest efficacy without significant trade-offs in terms of safety [43,44]. In line with these findings, the current European Society of Cardiology (ESC) endorses DES implantation and DCB angioplasty for the treatment of ISR (Class I) [34].

However, randomized trials comparing DCB vs. DES for ISR not infrequently revealed differences in angiographic and clinical outcomes that may reflect the influence of heterogeneous baseline ischemic risk conditions, ISR patterns, and procedural aspects, including device generation, lesion preparation, and intravascular imaging guidance (Table 1) [30,45,46,47,48,50,51,52,60,87]. In the ISAR-DESIRE 3 trial, DCBs and DESs were markedly more effective than plain balloons in terms of 6–8-month percentage diameter stenosis (*p* < 0.0001 for both comparisons), without significant difference between the two devices (*p* = 0.80) [49]. Nonetheless, at 1 year, a numerical trend towards a higher incidence of target lesion revascularizations was noted in the DCB group (22.1% vs. 13.5%; *p* = 0.09) [49]. This discrepancy may be attributable to the open-label design of the study and the fact that the presence of an additional stent layer in the DES group might have deterred operators from performing a repeat operation involving the implantation of another metal layer [49]. Later, RIBS IV compared the efficacy of DEBs versus everolimus-based DESs in the treatment of DES-ISR showing that DES was associated with an angiographic benefit 9 months after PCI compared with DCB, as evidenced by a significantly larger minimum lumen diameter (2.03 ± 0.7 mm vs. 1.80 ± 0.6 mm; *p* < 0.001) and a lower percentage of diameter stenosis (23 ± 22% vs. 30 ± 22%; *p* < 0.01) compared with DCB [47]. At 1 year, the primary composite endpoint of cardiac death, myocardial infarction, or target vessel revascularization was significantly lower in patients assigned to DES compared with those assigned to DCB (10% vs. 18%; HR 0.58; 95% CI 0.35–0.98; *p* = 0.04), mainly due to a significant reduction in target vessel revascularization (8% vs. 16%; *p* = 0.035) [84]. Target lesion revascularization was consistently lower with DES compared with DCB (4.5% vs. 13.0%; HR 0.33, 95% CI 0.14–0.79; *p* = 0.007), and no significant differences in cardiac death, myocardial infarction, and target lesion revascularization were observed between groups [47]. The 3-year results of RIBS IV did not reveal significant variations as DES continued to be associated with lower target lesion revascularization (7.1% vs. 15.6%; HR 0.43, 95% CI 0.21–0.87; *p* = 0.015) compared with DCB, without evidence of signals of harm [88]. In the DARE trial, DCB angioplasty was compared with DES implantation for the treatment of DES- and BMS-ISR, showing non-inferior results for 6-month in-segment minimal lumen diameter (1.74 ± 0.61 vs. 1.71 ± 0.51; P_noninferiority_ < 0.0001) [48]. Furthermore, no differences were found in 12-month major adverse clinical events and individual clinical endpoints [48]. Similar results were observed in the BIOLUX-RCT trial, in which PCI with DCB was compared with DES implantation for the treatment of DES- or BMS-ISR [60]. At 6-month angiography, DCB proved to be non-inferior to DES in terms of late lumen loss (absolute difference, −0.17 ± 0.52 mm; 97.5% CI −∞ to −0.01; *p* < 0.0001) at 6 months [60]. Furthermore, at 12 months, no differences were observed in terms of major adverse cardiac events and individual clinical endpoints [60].

**Figure 3:**
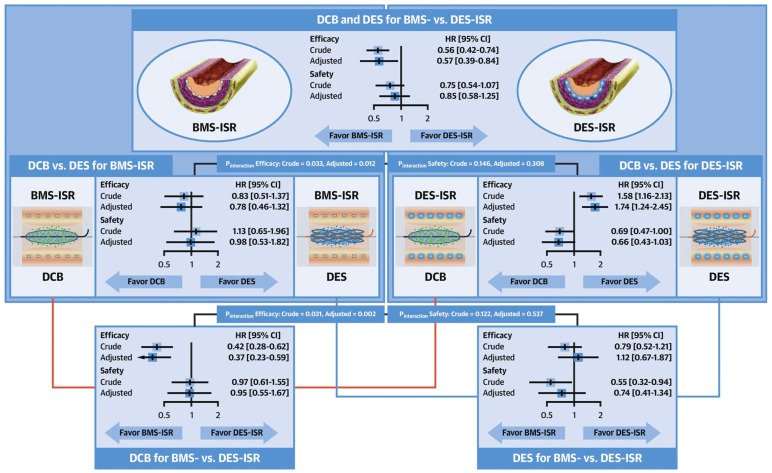
DCB vs. DES for BMS- and DES-ISR from all 10 available randomized trials. BMS = Bare metal stent; CI = confidence interval; DCB = drug-coated balloon; DES = drug-eluting stent; HR = hazard ratio. Efficacy refers to the primary efficacy endpoint of target lesion revascularization. Safety refers to the primary safety endpoint of a composite of death, myocardial infarction, or target lesion thrombosis.

Comprehensive results were obtained from the DAEDALUS individual patient data meta-analysis, in which 1976 patients undergoing DCB angioplasty or DES implantation in all 10 available randomized trials were compared at long-term follow-up [54]. At 3 years, DESs were moderately more effective than paclitaxel-eluting DCBs in reducing the rate of the primary efficacy endpoint of target lesion revascularization (HR 1.32; 95% CI, 1.02 to 1.70; *p* = 0.035) without statistically significant differences in the primary safety endpoint of a composite of all-cause death, myocardial infarction, or target lesion thrombosis (HR 0.80, 95% CI 0.58–1.09; *p* = 0.152) (Figure 2). Interestingly, a statistically significant interaction (*p* = 0.033) was found between the generation of DES used in the trial to treat ISR and the primary safety endpoint between the treatments. Specifically, DCB was associated with a lower incidence of all-cause death, myocardial infarction, or target lesion thrombosis compared with first-generation DES (HR 0.53, 95% CI 0.32–0.87; *p* = 0.012). In contrast, the primary safety endpoint was similar between DCB and second-generation DES (HR 1.06, 95% CI 0.71–1.60; *p* = 0.764) [54]. The DAEDALUS study also showed that the effectiveness between DCB and DES varies in relation to the type of stent previously implanted. In more detail, in the setting of BMS-ISR, there were no significant differences between DCB and DES in the primary efficacy endpoint of target lesion revascularization (9.2% vs. 10.2%; HR 0.83, 95% CI 0.51–1.37) and the primary safety endpoint (8.7% vs. 7.5%; HR 1.13, 95% CI 0.65–1.96). In contrast, in the setting of DES-ISR, target lesion revascularization was significantly higher after DCB angioplasty compared with repeat DES implantation (20.3% vs. 13.4%; HR 1.58, 95% CI 1.16–2.13), but the primary safety endpoint was numerically lower (9.5% vs. 13.3%; HR 0.69, 95% CI 0.47–1.00) (Figure 3) [89].

**Figure 4:**
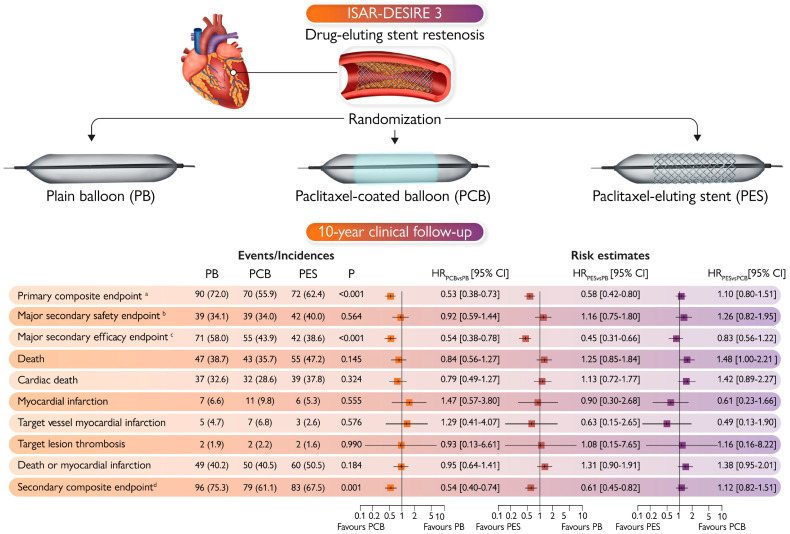
Ten-year outcomes following treatment of ISR. CI = Confidence interval; PB = plain balloon; PCB = paclitaxel-coated balloon; PES = paclitaxel-eluting stent; HR = hazard ratio. ^a^ Primary composite endpoint: Cardiac death, target vessel myocardial infarction, target lesion thrombosis, or target lesion revascularization. ^b^ Major secondary safety endpoint: Cardiac death, target vessel myocardial infarction, or target lesion thrombosis. ^c^ Major secondary efficacy endpoint: Target lesion revascularization. ^d^ Secondary composite endpoint: Death, myocardial infarction, target lesion thrombosis, or target lesion revascularization.

Recently, the 10-year clinical follow-up extension of the ISAR-DESIRE 3 trial showed no significant differences between DCB and DES in terms of the primary efficacy endpoint of target lesion revascularization (55.9% vs. 62.4%; *p* = 0.610) and the primary safety composite endpoint (Figure 4) [53]. However, although 10-year differences did not reach the threshold of statistical significance, an excess of death (9.3% vs. 20.9%; *p* = 0.028) and cardiac death (5.8% vs. 13.6%; *p* = 0.047) associated with DES compared with DCB was observed at 5-year landmark analyses [53]. Moreover, in the same study, an explorative competing risk analysis consistently showed a possible signal of harm associated with DES compared with DCB [53]. These results should be viewed in the context of a trial without statistical power for clinical endpoints and may be attributable to the use of first-generation DES [53]. Nevertheless, these alarming findings underline the need for further data about very long-term clinical outcomes following the treatment of DES-ISR with contemporary DESs.

## 6. Combined Interventional Strategies

Regardless of the strategy used, adequate lesion preparation plays a pivotal role in the treatment of ISR, especially when treated by DCB angioplasty. Indeed, DCB is not intended for lesion debulking but is only a carrier for delivering an antiproliferative medication through contact for 30 to 60 s with the vessel wall surface. Therefore, complete lesion length, gradual predilation with plain balloons of escalating size (balloon/vessel ratio 1.1:1) leading to a residual stenosis of <30%, without major dissection (≥type C) or coronary flow impairment (thrombolysis in myocardial infarction <3), seems to be relevant for DCB angioplasty [52,89]. However, even in the case of a DES-based strategy, several patterns of ISR, particularly those more resistant and diffuse, take advantage of thorough lesion preparation.

Some devices that have shown limited effectiveness as a standalone treatment for ISR, such as cutting or scoring balloons, improve the success of revascularization when employed in combined strategies [90,91]. Similarly, there is a growing interest in traditional and novel debulking devices, such as excimer laser coronary atherectomy and intravascular lithotripsy, for the treatment of ISR before DCB angioplasty and DES implantation [92,93].

### 6.1. Cutting and Scoring Balloons

Cutting balloons are conventional balloons equipped with 3–4 small blades attached longitudinally to create longitudinal incisions in the atherosclerotic plaque or fibrotic/calcified tissue, guaranteeing larger luminal diameters despite significantly lower inflation pressures than non-compliant or plain semi-compliant balloons [94]. Scoring balloons feature a similar technology with nitinol-based external helical cutting edge surrounding the balloon throughout its length [95,96].

Some trials have already demonstrated that the use of the cutting balloon alone compared with plain balloon angioplasty or DES implantation does not determine a reduction in the recurrence of ISR and the occurrence of major adverse cardiac events [97,98,99]. However, cutting and scoring balloons can be valuable devices for lesion preparation as they prevent the balloon from slipping out of the stent, allow for better penetration of the drug through the incisions in the plaque, and allow for a greater luminal gain in the case of resistant plaques [100,101]. The ISAR-DESIRE 4 randomized trial evaluated angiographic and clinical outcomes after lesion predilation with a scoring balloon in 252 patients with DES-ISR undergoing DCB angioplasty [90]. Adequate lesion preparation with scoring balloon followed by DCB angioplasty led to an advantage in terms of in-segment percentage diameter stenosis at 6–8 months compared with treatment with DCB angioplasty alone (35.0 ± 16.8% vs. 40.4 ± 21.4%; *p* = 0.047) [90]. At 1 year, there were no differences in the composite of major adverse cardiovascular events (4.0% vs. 3.4%; *p* = 0.73) and individual endpoints [90]. The ELEGANT trial showed similar results with a non-slip element balloon, characterized by the presence of three longitudinal nylon elements fixed proximally and distally to the balloon. In this trial, patients undergoing DCB angioplasty for ISR were randomly assigned to predilation with a non-slip element balloon or a high-pressure non-compliant balloon [91]. At 8 months, the primary endpoint of mean late lumen loss was similar between the two groups (0.28 ± 0.45 mm vs. 0.27 ± 0.38 mm, *p* = 0.75), though the use of the non-slip element balloon was associated with a reduction in balloon slippage (7.9% vs. 22.9%; *p* = 0.002) and geographic miss (6.9% vs. 21.9%; *p* = 0.002) [91].

Finally, a paclitaxel-coated scoring balloon, capable of combining the benefits of the scoring balloon with the loco-regional release of paclitaxel, showed promising results in a small, randomized trial, suggesting the need for further data [102].

### 6.2. Intravascular Lithotripsy

Intravascular lithotripsy is a recently introduced technique that is based on the locoregional emission of shock waves by multiple emitters positioned on a specific deliver catheter [103,104]. Once the target lesion has been reached, the balloon is inflated at low atmospheres, and one or more cycles of sonic waves are delivered to fragment the calcified plaque mass, favouring its subsequent dilation [103,104]. Despite favourable results in the treatment of de novo coronary artery disease, there are currently no randomized trials in the setting of ISR [103,105]. However, several case reports have demonstrated its feasibility for ISR, making it a therapeutic option for calcium debulking [106,107,108].

### 6.3. Rotational or Excimer Laser Atherectomy

Rotational atherectomy is an established procedure to ablate coronary plaques by the forward advancement of a rotating abrasive burr, which is particularly useful for the treatment of severely calcified lesions [109]. In early randomized trials, rotational atherectomy followed by plain balloon angioplasty for the treatment of ISR did not result in significant angiographic and clinical benefits compared with standalone plain balloon angioplasty [110]. Nevertheless, adjuvant rotational atherectomy before DCB angioplasty or DES implantation for the treatment of diffuse, severely obstructive, and recurrent ISR may be reasonable.

Excimer laser atherectomy is another atheroablative technique producing bursts of ultraviolet light energy that vaporizes, breaks, and debulks coronary plaques after absorption [111]. There are currently no randomized studies that support its routine use for the treatment of coronary artery disease. An early observational study showed that adjuvant excimer laser atherectomy for the treatment of BMS-ISR showed greater neointimal hyperplasia reduction compared with adjuvant rotational atherectomy [112]. However, 1-year target lesion revascularization was not significantly different between groups [112]. More recent small observational studies evaluated the angiographic outcomes of adjuvant excimer laser atherectomy in the treatment of ISR, demonstrating possible benefits in terms of luminal gain [113].

Nevertheless, whether adjuvant excimer laser or rotational atherectomy is associated with improved benefits when combined with DCB angioplasty and DES implantation is still undefined.

## 7. Bioresorbable Vascular Scaffold

Bioresorbable vascular scaffolds represent an attractive therapeutic approach for ISR as they provide mechanical support, release an antiproliferative medication during the post-implantation period, and gradually resorb over time. These favourable properties may theoretically prevent early elastic recoil, as for non-stent-based strategies, and potentially reduce recurrent ISR due to neoatherosclerosis and long-term thrombotic events by avoiding permanent implants [114,115]. However, first-generation bioresorbable vascular scaffolds showed inferior efficacy and safety for the treatment of de novo disease, and available studies in the setting of ISR did not meet the original expectations. Indeed, in an observational study comparing bioresorbable vascular scaffold implantation with DCB angioplasty or DES implantation for ISR, although angiographic and clinical results were not significantly different between bioresorbable scaffolds and DCB, DES was associated with reduced target lesion revascularization at 6–9 months [116]. In RIBS VI, patients prospectively assigned to bioresorbable vascular scaffolds for the treatment of ISR were compared with the historical control group of patients assigned to DCB and DES in the RIBS IV and RIBS V trials [116]. In this study, minimum lumen diameter after bioresorbable vascular scaffold implantation (1.87 ± 0.5 mm) was similar to that obtained after DCB (1.88 ± 0.6 mm; *p* > 0.05) but lower than that obtained after DES implantation (2.16 ± 0.7 mm; *p* < 0.001). Consistently, at 1 year, target lesion revascularization was similar between the bioresorbable vascular scaffold and DCB groups (11.3% vs. 10.4%; *p* = 0.86), but the incidence in patients who received DES implantation was lower than in patients who received bioresorbable vascular scaffolding (3.2% vs. 11.3%; *p* = 0.002) [116]. Although available data do not support this strategy, the results of studies with second-generation bioresorbable scaffolds may renew the interest in this class of devices.

## 8. Considerations on Medical Therapy

After treatment of ISR, it is essential to implement pharmacological therapy aimed at avoiding the main causes of failure, including recurrent ISR and stent thrombosis. ISR is more frequently associated with major ischemic risk factors and comorbidities, including diabetes mellitus, chronic kidney disease, and peripheral artery disease [9]. In addition, ISR is by definition more complex than de novo lesions and is associated with a higher prevalence of multivessel disease, multiple previous revascularizations, and previous myocardial infarction [9]. This clinical scenario may imply an increased risk of thrombotic events and adverse events in the case of nonadherence to medical therapy [117,118]. However, currently there is a paucity of data on the optimal antithrombotic therapy and duration following ISR treatment as ISR was a criterion for exclusion in most randomized trials, and when patients with ISR could be included, data of interest were underrepresented or unreported [119,120,121,122]. For these reasons, in contemporary clinical practice, patients receiving PCI for ISR are managed similarly to those undergoing PCI for de novo coronary artery disease. Dual antiplatelet therapy, de-escalation, and alternative chronic antithrombotic therapies can be considered based on the individual ischemic/bleeding risk profile [69,71,72,122,123,124,125,126,127,128,129,130,131]. Other pharmacological approaches involve, similarly to de novo coronary artery disease, the treatment of cardiovascular risk factors to mitigate the progression of the disease. In this context, the reduction of LDL cholesterol values plays a leading role, especially in the context of neoatherosclerosis [132,133]. A subanalysis of the FOURIER study demonstrated that in patients previously undergoing PCI, the administration of evolocumab not only resulted in a reduction in the risk of repeat revascularization compared with placebo (7.2% vs. 9.3%; HR 0.76, 95% CI 0.69–0.85), but also a reduction in the risk of major adverse cardiovascular events (HR 1.61, 95% CI 1.42–1.84; *p* < 0.0001) and major coronary events (HR 1.72, 95% CI 1.49–1.99; *p* < 0.0001) at 2 years [134]. Finally, the use of adjunctive anti-inflammatory or anti-proliferative medications has been suggested for patients with ISR, in particular for patients presenting with recurrent ISR. In the OSIRIS study, oral sirolimus resulted in a significant improvement in 6-month angiographic parameters, but this early benefit was attenuated at longer follow-up and concerns regarding potential side effects emerged [135,136]. Some studies are currently evaluating traditional anti-inflammatory drugs on top of optimal medical therapy for the treatment of recurrent ISR (NCT06090890). More interestingly, large-scale trials focusing on modern anti-inflammatory therapies in patients with coronary artery disease are underway. (NCT06118281) [137]. Whether these drugs will be effective for the treatment of coronary artery disease is uncertain, and the potential application in the setting of ISR warrants specific investigations.

## 9. Conclusions

Despite substantial advances in stent technology, ISR remains the primary cause of target lesion failure after PCI. The available evidence supports the use of DCB angioplasty and DES implantation as first-line therapies for ISR. Nonetheless, the choice between DES and DCB should be individualized based on clinical, anatomical, and technical factors. In this regard, intravascular imaging with IVUS and OCT can be useful for identifying the primary mechanisms leading to ISR and guiding the interventional strategy during PCI. Moreover, emerging combined approaches employing lesion modification with cutting/scoring balloons, intravascular lithotripsy, rotational atherectomy, or excimer laser in association with DCB or DES can be advantageous in the treatment of specific patterns of ISR. Further high-quality data are needed to define the differential effectiveness and safety of contemporary DCBs and DESs, the long-term clinical outcomes, and whether there are preferential clinical and anatomic conditions for the use of one device over the other.

## Figures and Tables

**Table 1:** Main randomized clinical trials on the treatment of ISR.

Trial	Year	Design	Sample Size	Treatments	Stent Type	Follow-Up	Primary Endpoint
**DES vs. PB**							
**ISAR-DESIRE [61]**	2005	Randomized, multicentre, open-label, 1:1:1	300	SES vs. PES vs. PB	BMS	Angiographic 6/8 months; Clinical: 12 months	Binary restenosis
**RIBS II [58]**	2006	Randomized, multicentre, open-label, 1:1	150	SES vs. PB	BMS	Angiographic and IVUS: 9 months; Clinical: 12 months	Binary restenosis
**CRISTAL [59]**	2012	Randomized, multicentre, open-label, 2:1	197	SES vs. PB	DES	Angiographic: 9–12 months	Late lumen loss
**DCB vs. PB**							
**Habara et al. [62]**	2011	Randomized, single-centre, single-blind, 1:1	50	PCB vs. PB	DES	Angiographic: 6 months; Clinical: 6 months	Late lumen loss
**PACCOCATH ISR I/II [63]**	2012	Randomized, multicentre, double-blind, 1:1	108	PCB vs. PB	BMS or DES	Angiographic: 6/9 months; Clinical: 12 months	Late lumen loss
**PEPCAD-DES [64]**	2012	Randomized, multicentre, single-blind, 2:1	110	PCB vs. PB	DES	Angiographic and clinical: 6 months	Late lumen loss
**Habara et al. [65]**	2013	Randomized, multicentre, open-label, 2:1	208	PCB vs. PB	BMS or DES	Angiographic: 6 months; Clinical: 6 months	Target vessel failure
**AGENT IDE [66]**	2024	Randomized, multicentre, open-label, 2:1	600	PCB vs. PB	BMS or DES	Clinical: 12 months	Target lesion failure
**DCB vs. DES**							
**PEPCAD II [51]**	2009	Randomized, multicentre, open-label, 1:1	131	PES vs. PCB	BMS	Angiographic 6 months; Clinical: 12 months	Late lumen loss
**ISAR DESIRE 3 [49]**	2013	Randomized, multicentre, open-label, 1:1:1	402	PES vs. PCB vs. PB	DES	Angiographic: 6/8 months; Clinical: 12 months	% Diameter stenosis
**PEPCAD China ISR [52]**	2014	Randomized, multicentre, single-blind, 1:1	215	PES vs. PCB	DES	Angiographic: 9 months; Clinical: 12 months	Late lumen loss
**RIBS V [67]**	2014	Randomized, multicentre, open-label, 1:1	189	EES vs. PCB	BMS	Angiographic: 6/9 months; Clinical: 12 months	Minimum lumen diameter
**SEDUCE [45]**	2014	Randomized, multicentre, open-label, 1:1	49	PCB vs. EES	BMS	Angiographic and OCT: 9 months; Clinical: 12 months	Uncovered struts
**RIBS IV [47]**	2015	Randomized, multicentre, open-label, 1:1	309	PCB vs. EES	DES	Angiographic: 6/9 months; Clinical: 12 months	Minimum lumen diameter
**TIS [50]**	2016	Randomized, multicentre, open-label, 1:1	136	PCB vs. EES	BMS	Angiographic: 12 months	In-segment late lumen loss
**DARE [48]**	2018	Randomized, multicentre, open-label, 1:1	278	PEB vs. EES	BMS or DES	Angiographic: 6 months; Clinical: 12 months	Minimum lumen diameter
**BIOLUX-RCT [60]**	2018	Randomized, multicentre, open-label, 1:2	229	EES vs. PCB	BMS or DES	Angiographic: 6 months; Clinical: 12 months	Late lumen loss; target lesion failure
**DCB vs. DCB**							
**RESTORE-ISR China [68]**	2018	Randomized, multicentre, open-label, 1:1	240	PCB vs. PCB	DES	Angiographic: 9 months	In-segment late loss
**Scheller et al. [69]**	2022	Randomized, multicentre, open-label, 1:1	101	SCB vs. PCB	DES	Angiographic: 6 months	Late lumen loss
**Han et al. [70]**	2023	Randomized, multicentre, open-label, 1:1	258	SCB vs. PCB	DES	Angiographic: 9 months	Late lumen loss
**REFORM [71]**	2023	Randomized, multicentre, single-blind, 1:1	201	PCB vs. BCB	BMS or DES	Angiographic: 6 months	Diameter stenosis (%)
**DES vs. DES**							
**ISAR-DESIRE II [30]**	2010	Randomized, multicentre, open-label, 1:1	450	SES vs. PES	DES	Angiographic: 6/8 months	Late lumen loss
**RESTENT-ISR [57]**	2016	Randomized, multicentre, open-label, 1:1	304	EES vs. ZES	DES	Angiographic and IVUS: 9 months; Clinical: 36 months	Neointima volume

BCB = Biolimus-coated balloon; BMS = bare metal stent; DES = drug-eluting stent; EES = everolimus-eluting stent; IVUS = intravascular ultrasound; OCT = optical coherence tomography; PB = plain balloon; PCB = paclitaxel-coated balloon; PES = paclitaxel-eluting stent; SCB = sirolimus-coated balloon; SES = sirolimus-eluting stent; ZES = zotarolimuse-luting stent.
